# Trypanosomatid Extracellular Vesicles as Potential Immunogens for Chagas Disease

**DOI:** 10.3390/ijms26041544

**Published:** 2025-02-12

**Authors:** Juliana Bernardi Aggio, Verônica Vitória Vedam, Líndice Mitie Nisimura, Rosiane Valeriano da Silva, Maria Izabel Lovo-Martins, Beatriz Santana Borges, Patrícia Alves Mörking, Michel Batista, Fabricio Klerynton Marchini, Sueli Fumie Yamada-Ogatta, Phileno Pinge-Filho, Samuel Goldenberg, Iriane Eger, Pryscilla Fanini Wowk

**Affiliations:** 1 Instituto Carlos Chagas, Fundação Oswaldo Cruz (FIOCRUZ-PR), Curitiba 81350-010, Brazil; juhaggio@gmail.com (J.B.A.); veronicavedam@gmail.com (V.V.V.); lindice.nisimura@fiocruz.br (L.M.N.); zane.valeriano@hotmail.com (R.V.d.S.); misabel@uel.br (M.I.L.-M.); biasborges@gmail.com (B.S.B.); pmorking@gmail.com (P.A.M.); samuel.goldenberg@fiocruz.br (S.G.); 2 Laboratório de Biologia Celular e Protozoologia, Universidade Estadual de Ponta Grossa, Ponta Grossa 84030-900, Brazil; 3 Grupo de Imunologia Molecular, Celular e Inteligência Artificial, Instituto Carlos Chagas, Fundação Oswaldo Cruz (FIOCRUZ-PR), Curitiba 81350-010, Brazil; 4 Laboratório de Imunopatologia Experimental, Departamento de Imunologia, Parasitologia e Patologia Geral, Centro de Ciências Biológicas, Universidade Estadual de Londrina, Londrina 86057-970, Brazil; pingefilho@uel.br; 5 Laboratório de Biologia Molecular de Microrganismos, Departamento de Microbiologia, Centro de Ciências Biológicas, Universidade Estadual de Londrina, Londrina 86057-970, Brazil; ogatta@uel.br

**Keywords:** extracellular vesicles, *Trypanosoma cruzi*, *Trypanosoma rangeli*, *Phytomonas serpens*, Chagas disease, cross-protection, proteomic, immunogenicity

## Abstract

Chagas disease remains a significant public health concern, with limited treatment options and an urgent need for novel preventive strategies. Extracellular vesicles (EVs) from *Trypanosoma cruzi* have been shown to modulate host immune responses, often favoring parasite persistence. In this study, we characterized EVs derived from the non-pathogenic trypanosomatids *Trypanosoma rangeli* and *Phytomonas serpens* and evaluated their potential as immunogens capable of inducing cross-protection against *T. cruzi* infection. Isolated EVs were characterized by Nanoparticle Tracking Analysis (NTA) and electron microscopy. A comparative proteomic analysis of EVs was performed using Mass Spectrometry-Based Proteomic Analysis (LC-MS/MS). The effects of EVs on immunomodulation and *T. cruzi* infection were assessed through in vitro and in vivo assays, using peripheral blood mononuclear cells (PBMCs) and BALB/c mice. The proteomic analysis identified shared proteins between the EVs of *T. rangeli*, *P. serpens*, and *T. cruzi*, including immunogenic candidates such as calpain-like cysteine peptidase and elongation factor 2. In vitro, pre-stimulation with the *T. rangeli* EVs reduced infection rates of the host cells by *T. cruzi*. In vivo, immunization with the EVs from *T. rangeli* and *P. serpens* led to a significant reduction in parasitemia in the BALB/c mice challenged with *T. cruzi*, though this did not translate into improved survival compared to controls. Interestingly, the EVs from *T. cruzi* also reduced parasitemia but did not confer protection against mortality. These findings suggest that while non-pathogenic trypanosomatid EVs exhibit potential immunogenic properties and can reduce parasitic load, their efficacy in preventing disease progression remains limited. Further research is needed to explore the mechanisms underlying these effects and to optimize EV-based strategies for protective immunity against Chagas disease.

## 1. Introduction

In the last decade, studies on extracellular vesicles (EVs) have highlighted their role in intercellular communication both within and between species. EVs are shed from cell membranes and carry proteins and nucleic acids from their source cells, which can modulate the response of target cells [1,2,3]. Based on their structure and content, EVs have been used as predictors of disease severity and progression [4], as well as potential immunogens in vaccine-based therapies [5,6,7].

Chagas disease is a neglected tropical health issue caused by the trypanosomatid *Trypanosoma cruzi*. It affects approximately 6–7 million people worldwide, leading to around 12,000 annual deaths, in addition to lifelong morbidity and disability [8,9]. The effectiveness of conventional treatments is very limited, and most individuals in the chronic phase carry the infection for life [10]. Furthermore, Chagas heart disease, a potentially fatal cardiomyopathy, is associated with severe cardiac inflammation [11]. The development of prophylactic vaccines and new treatments is urgently needed, but several challenges complicate the exploration of vaccines against Chagas disease, such as the genetic diversity of *T. cruzi* and its complex life cycle, which involves distinct stages of the parasite that express different antigens [12].

Protozoan EVs carry numerous molecules associated with host-cell interactions and virulence [13,14], so an immune response capable of neutralizing these factors may help control both the infection and its pathology. Trocoli-Torrecilhas et al. [15] demonstrated that immunizing BALB/c mice with EVs from *T. cruzi* followed by challenge with *T. cruzi* trypomastigotes resulted in increased parasite load and heart inflammation. This was accompanied by higher levels of Interleukin (IL)-10 and IL-4, as well as a lower survival rate compared to non-immunized animals [15]. Similarly, Lovo-Martins et al. [16] demonstrated that immunization of mice with *T. cruzi* EVs prior to infection modulated the host environment, supporting infection establishment. This led to reduced levels of inflammatory cytokines and nitric oxide, while favoring the formation of lipid bodies and Prostaglandin E2 (PGE2), which in turn increased parasitemia and cardiac parasitism [16]. These effects were also observed in vitro, where *T. cruzi* EVs induced higher intracellular Ca^2+^ levels and increased parasitism in host cells. Moreover, the EVs altered actin filaments and arrested the cell cycle, suggesting that *T. cruzi* EVs can modulate cell physiology to promote parasite survival [17]. These data indicated that *T. cruzi* EVs may not be a viable tool for immunization. However, considering that the protein components of EVs are potential immunogens in vaccine-based therapies for other diseases [6,18], our strategy to induce immunity against *T. cruzi* was to use EVs from related, but non-pathogenic, trypanosomatids—*Trypanosoma rangeli* and *Phytomonas serpens*—as both share antigens with *T. cruzi* [19,20,21,22,23].

*Trypanosoma rangeli* infection induces a humoral response in humans with cross-reactivity to *T. cruzi*, as both parasites share membrane antigens, particularly in their epimastigote forms, which show up to 60% similarity [24]. Moreover, several studies have demonstrated that immunization with *T. rangeli* can protect mice against *T. cruzi* infection, leading to reduced parasitemia, mild or absent heart injury, and increased survival rates [25,26]. Similarly, mice immunized either intraperitoneally or orally with *Phytomonas serpens*, a known phytoflagellate parasite, exhibited a significant reduction in parasitemia and increased survival [20,27]. These antigens are recognized by human sera and trigger nitric oxide-dependent protective immunity against experimental *T. cruzi* infection in susceptible BALB/c mice [20,28,29].

However, the mechanisms underlying these protective responses, including the potential role of *T. rangeli* and *P. serpens* EVs in cross-immunity, remain poorly understood. Could EVs from non-pathogenic trypanosomatids, which share antigenic molecules with *T. cruzi*, provide cross-protection against *T. cruzi* and mitigate infection? To explore the potential use of trypanosomatid EVs as immunogens against Chagas disease, we evaluated the effects of EVs from *T. rangeli* and *P. serpens* in comparison to those from *T. cruzi* in vitro and in vivo, alongside performing a proteomic analysis of the EVs.

Overall, our results suggest that EVs from *T. cruzi*-related parasites have antigenic potential. Despite only modest effects on cytokine modulation, costimulatory molecules, and *T. cruzi* infection in vitro, *T. rangeli* and *P. serpens* EVs contribute to reducing *T. cruzi* infectivity in BALB/c mice.

## 2. Results

### 2.1. Non-Pathogenic Trypanosomatids Shed Extracellular Vesicles

**Figure 1:**
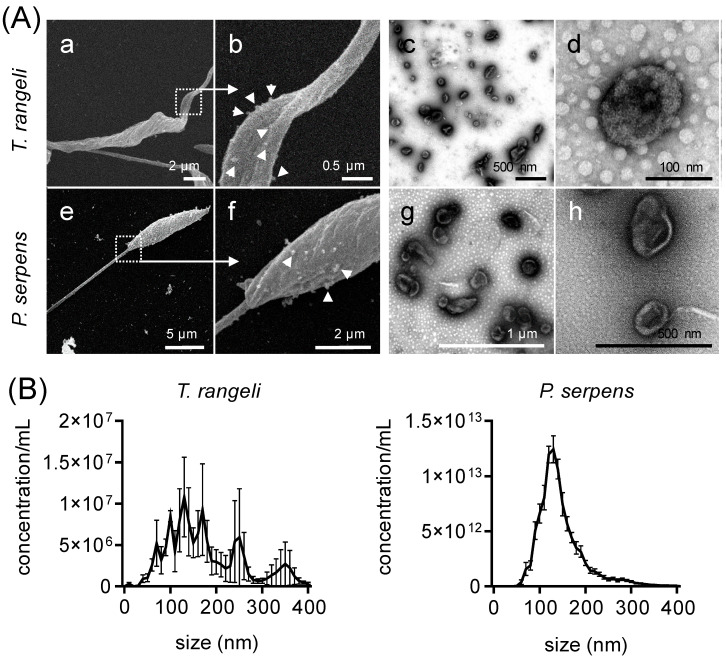
Extracellular vesicles of *Trypanosoma rangeli* and *Phytomonas serpens*. (**A**) Scanning electron microscopy of *T. rangeli* epimastigote (**a**,**b**) and *P. serpens* promastigote (**e**,**f**) shedding EVs (magnification = 4000–33,000×). Transmission electron microscopy of secreted EVs from these parasites in open field (**c**,**g**) and closed details (**d**,**h**). Triangles indicate EV shedding from cell membrane. (**B**) Concentration and size distribution of *T. rangeli* and *P. serpens* EVs. Bars represent ± standard error of the mean (SEM) from three independent experiments, each performed in duplicate.

First, we characterized the EVs derived from *T. rangeli* epimastigotes and *P. serpens* promastigotes using electron microscopy and NTA. Under nutritional stress, both parasites shed EVs from their entire body, including the flagellum (Figure 1A: panels a,b,e,f). The ultrastructure of these EVs (Figure 1A: panels c,d,g,h) revealed spherical or cup-shaped vesicles, which is consistent with the data obtained via NTA (Figure 1B). In the representative analysis, the *T. rangeli* EVs had a mean diameter of 176.7 ± 54.0 nm with a mode of 135.5 ± 35.8 nm, while the *P. serpens* EVs had a mean diameter of 149.3 ± 3.9 nm and a mode of 132.5 ± 3.5 nm (Figure 1B).

For the ELISA, we used EV samples derived from the *T. cruzi* and *T. rangeli* epimastigotes, shed under nutritional stress (without FBS), and purified using a commercial kit instead of ultracentrifugation. All the samples were analyzed by NTA and displayed a similar profile to the EVs purified by ultracentrifugation (Figure S1 in Supplementary Material). The *T. cruzi* EV had a mean of 241.4 ± 40.5 nm and a mode of 216.7 ± 34.8 nm, while the *T. rangeli* EV isolated using the kit had a mean size of 181.8 ± 14.3 nm and a mode of 143.45 ± 11.7 nm. We also characterized the EVs from uninfected Vero cells as a control for unrelated EVs, which had a mean size of 240.1 ± 54.3 nm and mode of 180.2 ± 34.2 nm (Figure S1 in Supplementary Material). The size range of the EVs obtained from all organisms was heterogeneous, indicating that our samples contained a mix of both large and small EVs. Given that no fractionation was performed, we will refer to the samples simply as extracellular vesicles (EVs).

### 2.2. T. rangeli EVs Cross-Reacted with Serum from Both Murine and Human T. cruzi-Infected Individuals

**Figure 2:**
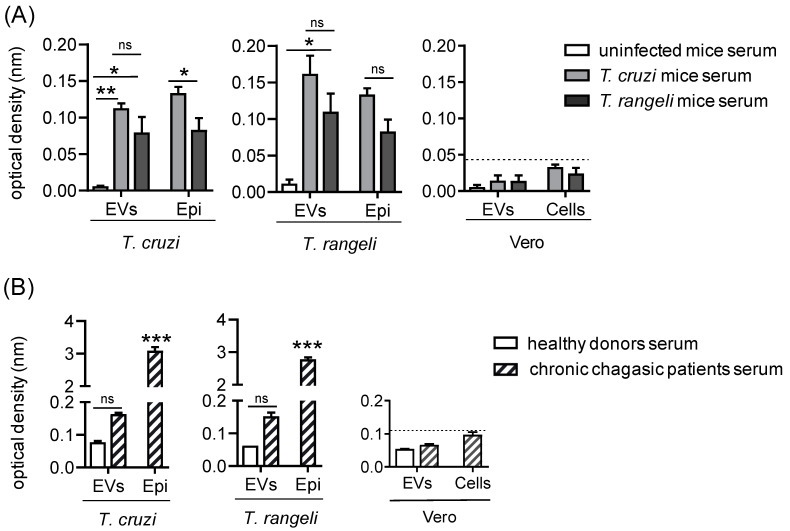
Cross-reacted antibodies against *T. cruzi* and *T. rangeli* EVs in the serum of infected mice and patients with chronic cardiac Chagas disease. (**A**) Cross-reactivity of EVs from *T. cruzi* epimastigotes (*T. cruzi* EVs), total protein extract of *T. cruzi* epimastigotes (*T. cruzi* Epi), EVs from *T. rangeli* epimastigotes (*T. rangeli* EVs), or total protein extract of *T*. *rangeli* epimastigotes (*T*. *rangeli* Epi) with pooled serum from three mice infected for 28 days with trypomastigotes of either *T. cruzi* or *T. rangeli*. Serum from uninfected mice served as control. (**B**) Reactivity of the same antigens from panel (**A**) with pooled serum from five chronic Chagas disease patients having the cardiac form. Serum from healthy donors was used as control. Bars represents the standard deviation (SD) of two independent experiments. The asterisk (*) indicates a statistically significant difference between the reaction of uninfected and parasite-infected mice serum (**A**), and between healthy donor and patient serum (**B**), for each antigen. ns = not significant, * *p* < 0.05, ** *p* < 0.01, and *** *p* < 0.001.

To investigate the potential of non-pathogenic parasite-derived EVs as an immunogenic tool against Chagas disease, we tested their immunogenicity and cross-reactivity with the total *T. cruzi* proteins and EVs. The serum from infected mice (Figure 2A) reacted to both the *T cruzi* and *T. rangeli* EVs at titers of up to 1:100, regardless of whether the mice were infected with *T. cruzi* or *T. rangeli* (ns, *p* > 0.05), indicating cross-reactivity. In contrast, the serum from uninfected mice did not recognize the EVs. As expected, both the infected mouse sera reacted with the total epimastigotes extracts. However, none of the mouse sera recognized the Vero cell EVs or the total cell extracts (cut-off = 0.4) (Figure 2A).

These findings were confirmed using human sera (Figure 2B), where the serum from chronic *T. cruzi*-infected patients also recognized both the *T. cruzi* and *T. rangeli* EVs, with titers up to 1:500. However, this reaction was nonspecific, as the serum from healthy donors also recognized some of the EV antigens (ns, *p* > 0.05). This nonspecificity was not due to poor serum quality, as the *T. cruzi* and *T. rangeli* epimastigote extracts showed strong reactions (Figure 2B). Given that the sera were polyclonal, and no recognition was observed for Vero cell antigens (cut-off = 0.11), it is suggested that only parasite-derived EVs reacted with Chagas disease patients serum above this threshold.

In summary, these data indicate that *T. rangeli* EVs carry antigens that can be recognized by antibodies in both murine and human-infected sera, with a response similar to that observed with *T. cruzi* EVs. To further investigate these similarities, we performed a proteome analysis to identify antigenic proteins present in the EVs of non-pathogenic parasites.

### 2.3. Common Proteins Shared Among Trypanosomatid EVs

**Figure 3:**
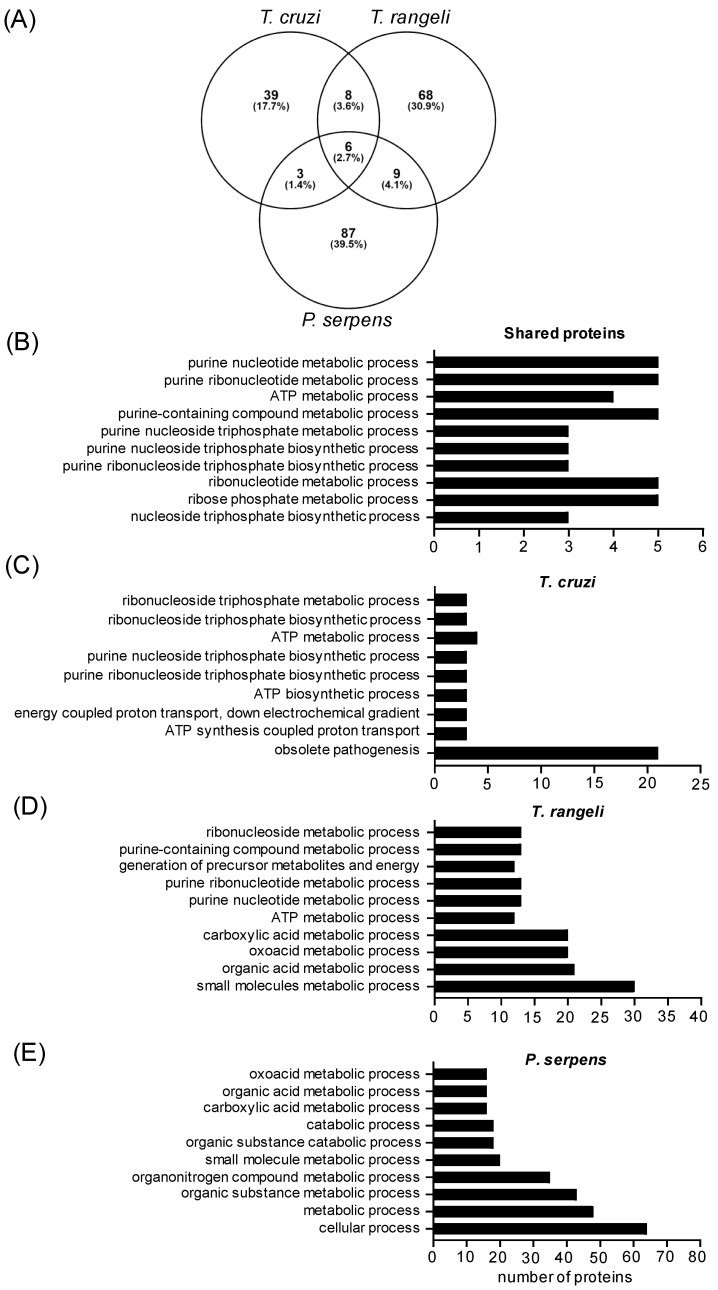
Identified EV proteins from *T. cruzi* trypomastigotes, *T. rangeli* epimastigotes, and *P. serpens* promastigotes. (**A**) Venn diagram with the percentage of common and distinct proteins by species. (**B**) Top 10 enriched biological process GO terms, ranked according to FDR, for proteins shared by at least 2 species, (**C**) for all identified proteins in the EVs of *T. cruzi* trypomastigotes, (**D**) for *T. rangeli* epimastigotes, and (**E**) for *P. serpens* promastigotes.

Since mammals are infected by *T. cruzi* trypomastigotes, our goal was to identify cross-reactive antigens in EVs from epimastigotes of *T. rangeli* and promastigote forms of *P. serpens*, and we performed a cross-analysis of EVs from these three sources using LC–MS/MS. All the MS/MS spectra were searched against databases for the three species, which enriched the identifications. Using this approach, we identified 105 proteins in *P. serpens* EVs, 91 in *T. rangeli* EVs, and 56 in *T. cruzi* EVs. After performing orthology analysis, we found 194 distinct proteins, with 26 shared proteins (Figure 3 and Table 1). The six proteins identified in all three species’ EVs were elongation factor 2, calpain-like cysteine peptidase, tryparedoxin peroxidase, enolase, alpha tubulin, and P-type H+-ATPase.

The top 10 GO terms related to the biological process for all species, and each individual species is presented in Figure 3B and Figure 3C–E, respectively. A total of 26 proteins were shared by at least two species. The enriched GO terms for this group were related to nucleoside and purine metabolic processes (Figure 3B). In the *T. cruzi* EVs, the GO term “obsolete pathogenesis” (GO:0009405) refers to processes that enable an organism to induce an abnormal, often detrimental state in another organism (www.ebi.ac.uk, accessed on 24 October 2024). A complete list of the proteins identified for the first time in *P. serpens* and *T. rangeli* EVs, along with the annotated *P. serpens* database and full list of enriched GO terms, is available in Supplementary Material—Table S1.

Despite the majority of identified proteins being exclusive to each parasite’s EVs (88.1%), we searched for predicted epitopes and transmembrane (TM) domains in the data to better understand the cross-immunoreactivity observed in the ELISA. Using the tools available on the TriTrypDB site, we found 11 proteins with TM domains in *T. rangeli* EVs, 5 proteins with TM domains in *T. cruzi* EVs (Table 2), and 7 proteins with predicted epitope sequences (Supplementary Material—Table S2)—primarily from trans-sialidase and heat shock protein families. For *P. serpens* EVs, no proteins meeting these criteria were found, due to the limited annotations in the current *P. serpens* database.

Once the described proteins (Table 2) are exposed in the membrane of the parasite’s shed EVs, and some of them also have predicted epitopes, these characteristics reinforce the potential of EV structures from non-pathogenic parasites as immunogens. Thus, we next present the investigations of the EVs’ biological functions in both in vitro and in vivo models.

### 2.4. Trypanosomatid EVs Are Inflammatory but Mildly Modulate CD80 Expression

**Figure 4:**
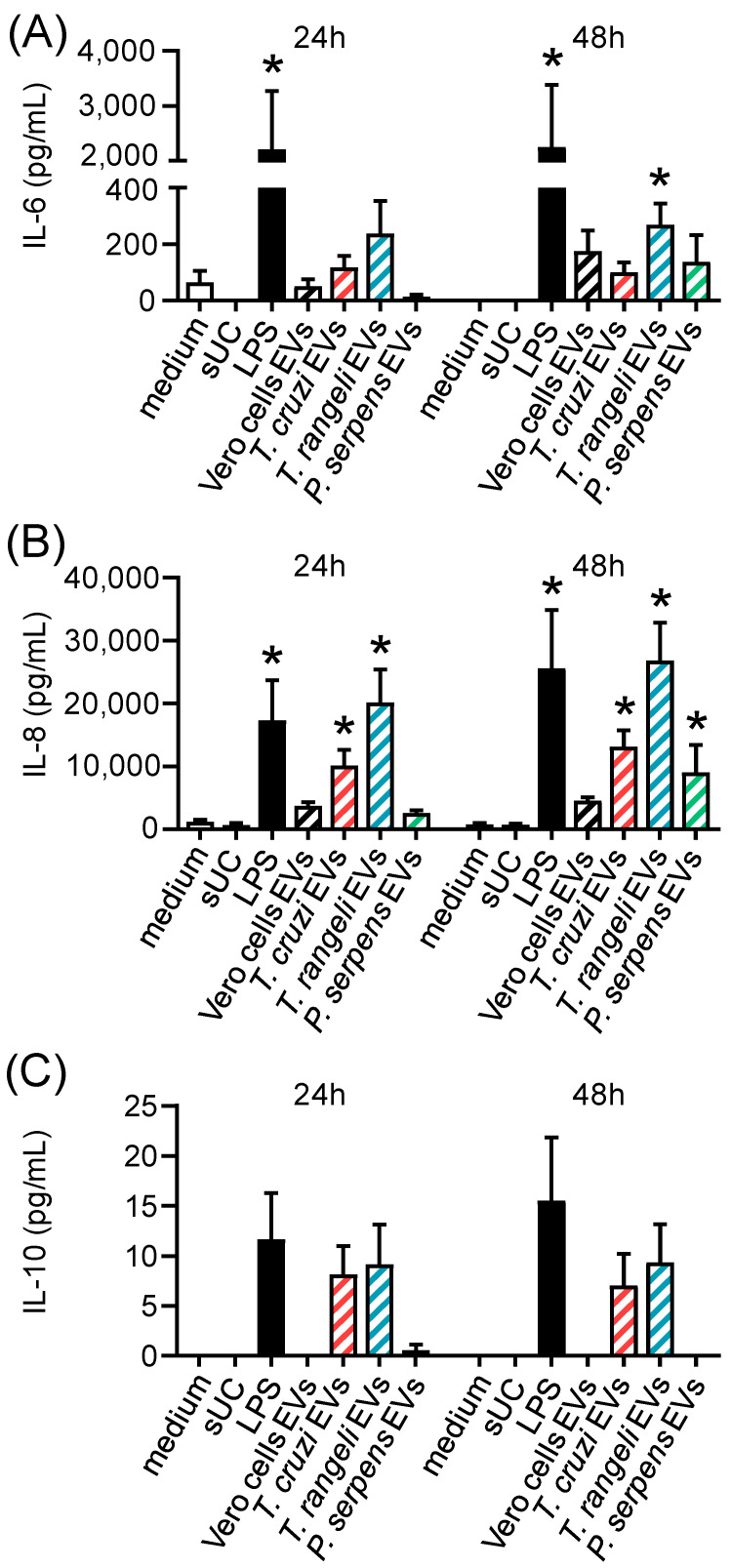
Secretion of inflammatory cytokines by PBMCs stimulated with *T. cruzi*, *T. rangeli*, and *P. serpens* EVs. Levels of IL-6 (**A**), IL-8 (**B**), and IL-10 (**C**) were measured in the supernatants of PBMCs 24 or 48 h after stimulation with medium, supernatant from ultracentrifugation (sUC), 1000 ng of EVs from Vero cells, LPS (100 ng/mL), 1000 ng of EVs from *T. cruzi* trypomastigotes, 1000 ng of EVs from *T. rangeli* epimastigotes, or 1000 ng of EVs from *P. serpens* promastigotes. Bars represent the standard error of the mean (SEM). Data from two independent experiments are shown (*n* = 3–6). The asterisk (*) indicates statistically significant difference between the medium and all other stimuli.

To evaluate the immunomodulatory potential of trypanosomatid EVs, we performed an in vitro assay using PBMCs from healthy individuals. These cells were stimulated with EVs from *T. cruzi*, *T. rangeli*, or *P. serpens* for 24 or 48 h. Supernatant from ultracentrifugation (sUC), EVs from Vero cells, and LPS were used as controls. Cytokine levels in the supernatant indicated that trypanosomatid EVs can induce the release of inflammatory cytokines, such as IL-6 (Figure 4A), with a significant increase in IL-6 levels after 48 h of stimulation with *T. rangeli* EV. The levels of IL-8, an important cell chemoattractant, were elevated by *T. cruzi* and *T. rangeli* EVs after 24 h and by all parasite EVs after 48 h (Figure 4B). IL-10 release was also increased following stimulation with *T. cruzi* and *T. rangeli* EVs, though the change was not statistically significant (Figure 4C).

**Figure 5:**
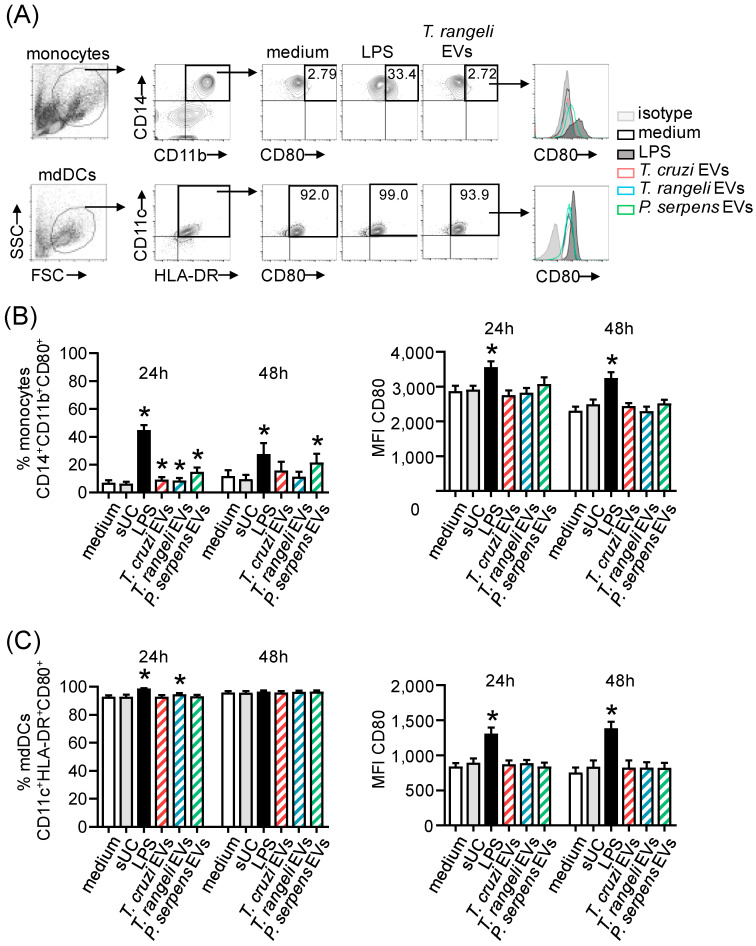
Mild modulation of CD80 expression in monocytes and mdDCs by *T. cruzi*-related parasite EVs. (**A**) Flow cytometry plots showing the frequency of CD14^+^CD11b^+^CD80^+^ monocytes and CD11c^+^HLA-DR^+^CD80^+^ mdDCs, as well as the fluorescence intensity of CD80 within these populations, 24 h after stimulation with medium, LPS (100 ng/mL), or 1000 ng of EVs from *T. rangeli* epimastigotes. This time point and these stimuli were selected to represent the overall results. Isotype control antibodies were used as a negative fluorescence control to set the gates and histograms. (**B**) Frequency and mean fluorescence intensity (MFI) of CD80 gated in the CD14^+^CD11b^+^ population, 24 and 48 h after stimulation with medium, supernatant from ultracentrifugation (sUC), LPS (100 ng/mL), 1000 ng of EVs from *T. cruzi* trypomastigotes, 1000 ng of EVs from *T. rangeli* epimastigotes, or 1000 ng of EVs from *P. serpens* promastigotes. (**C**) The same analysis as in (**B**) was performed for CD80^+^ mdDCs gated in the CD11c^+^HLA-DR^+^. Bars represent the standard error of the mean (SEM). Data from two to three independent experiments are shown (*n* = 8–12). The asterisk (*) indicates a statistically significant difference between the medium and all other stimuli.

The expression of CD80, one of the costimulatory molecules involved in antigen presentation and T-cell activation, was also evaluated on the surface of monocytes and dendritic cells after EV stimulation by flow cytometry. Single-color-stained samples were used for instrument compensation, and control isotypes were also tested. However, one limitation of the analysis was that Fluorescence Minus One (FMO) control analysis could not be performed. Figure 5A shows the gating strategy used to identify monocytes (CD14^+^CD11b^+^) and monocyte-derived dendritic cells (mdDCs—CD11c^+^HLA-DR^+^).

Stimulation with EVs from all parasites led to an increase in the frequency of CD14^+^CD11b^+^CD80^+^ cells after 24 h. After 48 h, a higher percentage of CD80^+^ monocytes was observed only in *P. serpens* EV-stimulated cultures (Figure 5B, left panel). The frequency of CD11c^+^HLA-DR^+^CD80^+^ mDCs was significantly increased in cultures stimulated with *T. rangeli* EVs after 24h (Figure 5C, left panel). However, the expression levels, measured by the mean fluorescence intensity (MFI), were not significantly affected in the evaluated cells (Figure 5B,C, right panel).

### 2.5. Prior Stimulation with Trypanosomatid EVs In Vitro Reduces T. cruzi Infection

**Figure 6:**
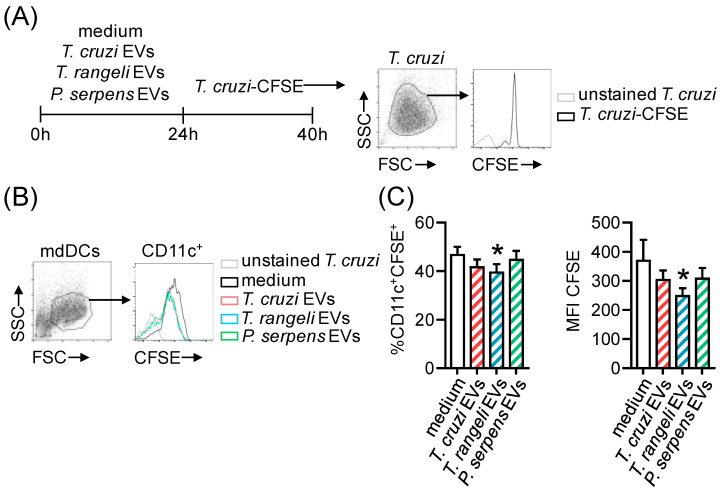
*T. cruzi* infection in CD11c^+^ mdDCs is modulated by pre-treatment with *T. rangeli* EVs. (**A**) CD11c^+^ mdDCs were stimulated for 24 h with medium, 1000 ng of EVs from *T. cruzi* trypomastigotes, 1000 ng of EVs from *T. rangeli* epimastigotes, or 1000 ng of EVs from *P. serpens* promastigotes, and then infected with *T. cruzi* trypomastigotes (10 MOI) labeled with CFSE. CD11c^+^ mdDCs infected with *T. cruzi* (without CFSE labelling) were used as negative control. (**B**) Representative histogram showing the fluorescence intensity of CFSE inside CD11c^+^ mdDCs after *T. cruzi* infection in cells pre-stimulated with parasite EVs. (**C**) Frequency of CD11c^+^CFSE^+^ mdDCs and the mean fluorescence intensity (MFI) of CFSE in this population after EV stimulation and *T. cruzi*-CFSE infection. Bars represent the standard error of the mean (SEM). Three independent experiments are shown (*n* = 10–14). The asterisk (*) indicates a statistically significant difference between the medium and *T. rangeli* EV stimulation (panel **C**).

Our next question was whether EV stimulation affects infection in targets cells. Monocyte-derived dendritic cells (mdDCs) from healthy individuals were stimulated with EVs from *T. cruzi*, *T. rangeli*, or *P. serpens.* Twenty-four hours later, *T. cruzi*-CFSE^+^ trypomastigote forms were added to the culture (Figure 6A), and the frequency of infected cells (CFSE^+^) was evaluated 16 h later (Figure 6B,C). Our results show that prior stimulation of mdDCs with *T. rangeli* EVs was able to reduce the frequency of infected cells (Figure 6C, left panel) and also the intracellular parasite load, expressed by MFI CFSE levels (Figure 6C, right panel), compared to cells without EV stimulation (the medium).

### 2.6. T. cruzi-Related Parasite EV Immunization Protects Mice from T. cruzi Infection

**Figure 7:**
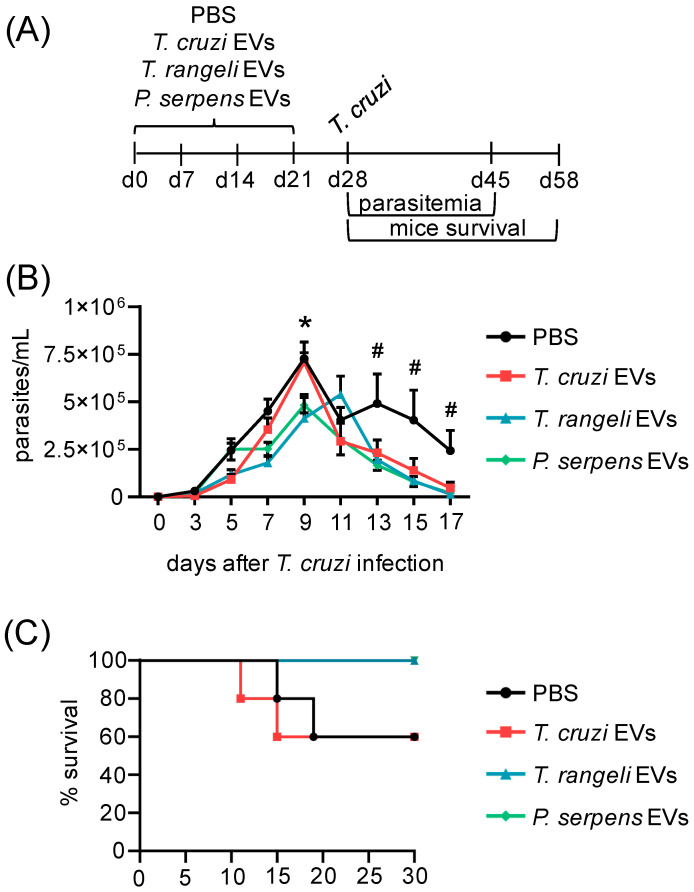
Immunization with *T. rangeli* and *P. serpens* EVs increases mice survival after *T. cruzi* infection. (**A**) BALB/c mice were inoculated four times with medium, 5 µg of EVs from *T. cruzi* trypomastigotes, 5 µg of EVs from *T. rangeli* epimastigotes, or 5 µg of EVs from *P. serpens* promastigotes, followed by a challenge with *T. cruzi*. The parasitemia in the blood (**B**) and animal survival rates (**C**) were monitored up to 30 days post-infection (dpi). Bars represent the standard error of the mean (SEM). Data from one of two independent experiments are shown. Three to five animals per group were used in each experiment. * indicates a statistically significant difference between *T. rangeli* or *P. serpens* EV groups and the PBS control, and **#** indicates a statistically significant difference between the trypanosomatid EV groups and the PBS control.

To test our findings in vivo and evaluate whether trypanosomatid EV immunization could alter *T. cruzi* infection, we immunized BALB/c mice with four doses of EVs from *T. cruzi*, *T. rangeli*, or *P. serpens*, administered at 7-day intervals. One week after the final EV immunization, the animals were challenged with *T. cruzi* blood trypomastigotes (Figure 7A). The parasitemia peak in PBS, *T. cruzi*, and *P. serpens* EV-treated animals occurred at 9 dpi, while the peak for *T. rangeli* EV-treated animals occurred at 11 dpi (Figure 7B). Pre-immunization with *P. serpens* EVs significantly reduced parasitemia (Figure 7B). Although no statistically significant difference was observed, the survival data supported the parasitemia results, showing that 100% of the animals immunized with *T. rangeli* or *P. serpens* EVs were alive after 30 days, compared to a 60% survival rate in the PBS and *T. cruzi* EV-immunized groups, where mortality began after the peak of parasitemia (Figure 7C).

## 3. Discussion

In this study, we characterized extracellular vesicles (EVs) from non-pathogenic trypanosomatids (*T. rangeli* and *P. serpens*) and evaluated their in vitro and in vivo effects to determine whether these EVs could provide cross-protection against *T. cruzi* infection. EVs are known to carry a heterogeneous cargo, including small metabolites, proteins, lipids, and nucleic acids. This cargo can vary depending of the source, as well as the physiological or pathological state, which in turn mediates distinct cellular communications and biological effects [2,3].

Unlike *T. cruzi* EVs, the characteristics of *T. rangeli* and *P. serpens* EVs are not well understood. Therefore, we analyzed and, for the first time, described the physical properties and protein cargo of EVs shed by these trypanosomatids. Similarly to *T. cruzi,* under nutritional stress, *T. rangeli* and *P. serpens* shed spherical or cup-shaped EVs throughout the body, primarily from the flagellum [30,31]. Using the same methodology, we observed that the size of *T. cruzi* epimastigote-derived EVs were similar to those previously described [17,32], but the EV population was larger than that found for *T. rangeli* and *P. serpens* EVs.

The first report on *T. cruzi* EVs dates back to 1979, when membrane vesicles from epimastigote forms were isolated and analyzed. The study showed that antibodies raised against these purified vesicles were able to agglutinate *T. cruzi* epimastigotes [33], suggesting the antigenic properties of EVs. In 1991, Gonçalves et al. proposed that *T. cruzi* trypomastigotes spontaneously shed polypeptides into the medium as plasma membrane vesicles, which could contain proteins involved in host cell invasion, such as the Tc-85 and 85 kDa surface glycoproteins [34].

It is well established that EVs have immunomodulatory effects. One such mechanism was described for EVs released by THP-1 cells infected with *T. cruzi*, which interacted with the Toll-like receptor (TLR2), thereby increasing the susceptibility of THP-1 cells to *T. cruzi* infection. This interaction activated the NF-κB pathway, leading to the production of proinflammatory cytokines (IL-1β and IL-6) that sustained the inflammatory response induced by *T. cruzi* infection. Additionally, *T. cruzi*-infected THP-1-derived EVs were found to carry parasite proteins [35].

In a similar study, another group demonstrated that microvesicles (MVs) derived from tissue-culture and metacyclic trypomastigotes of *T. cruzi* could interact with THP-1 cells and fuse with the THP-1 membrane, facilitating parasite entry [13]. Serum from chronic Chagas disease patients having indeterminate and cardiac forms detected antigens from these MVs, which had originated from the interaction between *T. cruzi* infective forms and THP-1 cells. In contrast, no serum reactivity was observed with MVs derived from interactions with non-infective epimastigote forms. Moreover, the pattern of molecules detected by sera from indeterminate versus cardiac forms of Chagas disease was distinct, suggesting the presence of specific markers in MVs that could help differentiate between forms of the Chagas disease [13].

In addition, another study found that *T. cruzi* metacyclic trypomastigotes induced host blood cells and THP-1, both in vitro and in vivo, to release MVs involved in inhibiting complement-mediated lysis, through interference with C3 convertase cleavage on the surface of *T. cruzi,* and increased cell invasion and parasitemia via TGF-β carried in the MVs [36].

The in vivo effects of *T. cruzi* EVs have also been well documented in the literature. BALB/c mice pretreated with *T. cruzi* trypomastigote-derived membrane vesicles, when challenged with infective forms, exhibited exacerbated heart parasitism and inflammation. This was accompanied by increased IL-10 and IL-4 production, which preceded mortality in the animals [15]. Similarly, EVs from four *T. cruzi* strains (Y, Colombiana, CL-14, and YuYu) were able to induce differential expression of TNF-α, IFN-γ, IL-6, IL-10, and NO via TLR2 in splenocytes from C57BL/6 mice chronically infected with *T. cruzi* [31]. Furthermore, a lower percentage of MHC-II-positive macrophages was observed in C57BL/6 mice infected with *T. cruzi*, which had been previously inoculated with *T. cruzi* EVs [16]. When PBMCs from healthy donors were stimulated in vitro with trypanosomatids EVs, we observed modulation of IL-6, IL-8, and IL-10 secretion in the culture supernatant, as well as a mild modulation on CD80 expression. This finding reinforces the role of EVs as antigenic stimuli in both early and late events of the immune response.

Moreover, EVs from PBMCs stimulated with *T. cruzi* and EVs from the plasma of chronically infected Chagas disease patients both induced a differential inflammatory gene expression profile, cytokine release, and nitric oxide production in THP-1 cells [37]. Mucin-associated surface proteins (MASP family) present in *T. cruzi* EVs and those from chronic Chagas disease patients elicited a stronger antibody response against the MASP signal peptide (SP) region. Immune complexes formed in the sera of these patients consisted of *T. cruzi* EVs carrying MASP SP, and these EVs were shown to inhibit complement-mediated lysis [38]. Overall, EVs from chronic Chagas disease patients increased IFN-γ and IL-17 production in previously stimulated THP-1 cells, suggesting a more inflammatory environment conductive to the establishment of chronic disease [39].

Under the conditions described here, sera from both murine and human *T. cruzi*-infected individuals showed antigenic reactivity against EVs from both *T. rangeli* and *T. cruzi* epimastigote forms, indicating cross-reactivity. However, the human serum detection was non-specific to trypanosomatid EVs, regardless of the chronic form (cardiac or indeterminate), or even to acute Chagas disease serum.

To better understand this cross-reaction and identify which EV components might be modulating the immune response, we performed a comparative proteomic analysis of *T. rangeli* epimastigote EVs, *P. serpens* promastigote EVs, and EVs from the infective *T. cruzi* trypomastigote form. Among the six proteins identified in common across all the three species, in addition to cytoskeleton and basic metabolism, we found immunogenic candidates such as calpain-like cysteine peptidase and elongation factor 2.

Moreover, one of the shared proteins identified in *T. cruzi* and *T. rangeli* EVs was GP63, a metalloprotease with a glycosylphosphatidylinositol (GPI)-anchor to the membrane. Since antiserum from recombinant GP63-immunized mice has been shown to inhibit host cell infection by *T. cruzi* [40,41], the presence of GP63 in *T. rangeli* EVs could be a key component explaining the protection observed in *T. rangeli* EV-immunized mice. A similar protective effect has been described with GP90 and GP82 released by *T. cruzi* G strain metacyclic forms in both EVs and soluble protein fractions, which were shown to impair parasite invasion of Hela cells [42]. Our main limitation in the proteomic analysis was the quality of the genomic and proteomic annotations for *T. rangeli* and *P. serpens*. Future revisions of these data may provide valuable insights to help explain the cross-reaction between EVs from these protozoa.

Since trypanosomatid-derived EVs share relevant proteins that could be involved in immunomodulation, we aimed to evaluate whether EV stimulation could also affect the in vitro infection rate of human cells. When mdDCs were stimulated with trypanosomatid EVs and then cultured 24 h later in the presence of *T. cruzi*-CFSE^+^ of trypomastigotes, we assessed the frequency of infected cells and the intracellular parasite load 16 h post-infection. Our results showed that pre-stimulation with *T. rangeli* EVs reduced both the frequency of CD11c^+^ cells infected with *T. cruzi* and the intracellular parasite load (measured by CFSE MFI), suggesting a potential protective effect. It is important to note that after 16 h, the reduced CFSE intensity indicates a lower number of internalized parasites and no parasite proliferation. Interestingly, pre-stimulation with *T. cruzi* EVs did not affect *T. cruzi* infection. In contrast, stimulation of bone marrow-derived macrophages from C57BL/6 mice with *T. cruzi* EVs for 24 h increased both the parasite infection rate and the number of intracellular parasites [16].

Lastly, our in vivo results showed that EVs from *T. rangeli* and *P. serpens* reduced parasitemia in BALB/c mice infected with *T. cruzi*, suggesting a potential protective effect of these vesicles. However, *T. cruzi* EVs also reduced parasitemia, which may initially seem contradictory given the previously reported deleterious effects of these vesicles [15,16]. It is important to note that in our study, the animals were immunized with four doses of EVs, and the challenge was performed 28 days after the first immunization. This reduction in parasitemia could be linked to an initial host immune response triggered by the vesicle components, such as immunogenic proteins, including trans-sialidases and other surface proteins known to stimulate inflammatory responses. Nonetheless, despite the decrease in parasitemia, immunization with *T. cruzi* EVs did not confer significant protection against mortality, similar to the outcome observed in the control group. However, these results raise important questions, such as the level of heart parasitism, cardiac tissue inflammation, iNOS activity, and cytokine expression. Future studies will be crucial to understanding the mechanisms underlying these protective effects and will help assess the potential use of trypanosomatid-derived EVs as immunogens for the prevention of Chagas disease.

## 4. Conclusions

These findings highlight the complexity of the immune response induced by *T. cruzi* EVs, which initially reduce parasitic load but fail to provide lasting protection against infection progression and mortality. In contrast, EVs from non-pathogenic trypanosomatids show potential immunogenic properties and can reduce parasitic load, though their ability to prevent disease progression remains limited. Future studies are needed to better understand the mechanisms underlying this paradoxical effect and to explore whether targeted modulation of EV components could enhance the protective response.

## 5. Materials and Methods

### 5.1. Cells

Vero cells (ATCC C1008) were maintained in RPMI-1640 medium with 2 mM L-glutamine (Lonza, Basel, Switzerland), supplemented with 10% fetal bovine serum (FBS; Gibco, Grand Island, NY, USA) and 25 μg/mL gentamicin (Gibco) at 37 °C in a 5% CO_2_, humidified atmosphere. Peripheral blood was obtained by intravenous puncture from healthy volunteers (6 to 12 samples per assay, aged 21–50 years, regardless of gender, and without clinical evidence of disease) upon written consent. The procedures were approved by the Fundação Oswaldo Cruz (FIOCRUZ) research ethics committee under the protocol number CAAE: 49931415.7.1001.5248.

Peripheral blood mononuclear cells (PBMCs) were isolated using Histopaque density 1.077 g/mL (Lonza, Basel, Switzerland). CD14^+^ monocytes were sorted using the MACS system (Miltenyi Biotec, Bergisch Gladbach, Germany) according to the manufacturer’s instructions and seeded at 5 × 10^5^ cells/mL in a 150 cm^2^ cell culture flask containing 20 mL of RPMI-1640 medium supplemented with 10% FBS, 25 µg/mL gentamicin, 12.5 ng/mL recombinant human GM-CSF (PeproTech, Rocky Hill, NJ, USA), and 25 ng/mL recombinant human IL-4 (PeproTech, Rocky Hill, NJ, USA). The cells were incubated for 7 days at 37 °C in a 5% CO_2_, humidified atmosphere. On the third day of incubation, fresh supplemented medium was added to the cell culture. Differentiation of human monocyte-derived dendritic cells (mdDCs) was confirmed by flow cytometry (CD11c^^+^/high^CD14^^+^/low^). Cell viability was determined by Trypan blue exclusion counting. The FBS used in this study was EV-depleted (centrifuged at 100,000× *g* for 16 h at 4 °C) and filtered through a 0.22 μm filter.

### 5.2. Parasites

Blood trypomastigotes of *Trypanosoma cruzi* Y strain [43] were maintained by weekly intraperitoneal inoculation of Swiss mice (2 animals per challenge assay), with 2 × 10^5^ trypomastigotes (ethics committee protocol 051.2023, Universidade Estadual de Londrina). Infected mice at the peak of parasitemia (7 days post-infection) were euthanized, and the blood was collected by cardiac punction to obtain bloodstream trypomastigotes. After determining the total number of parasites, some of the cells were used for mouse infection, while the remaining parasites were used to infect Vero cells (MOI 10) cultured in RPMI-1640 medium supplemented with 10% FBS. Every 3 to 4 days, trypomastigotes from the infected Vero cells were harvested for EV purification, for in vitro infection, or to infect new Vero cell culture.

Epimastigotes of *T. cruzi* Y strain were cultured at 28 °C in liver infusion tryptose (LIT) medium, supplemented with 10% FBS and 50 IU/mL penicillin-streptomycin (Sigma-Aldrich, St. Louis, MO, USA). Epimastigotes of *Trypanosoma rangeli* Choachi strain [44], provided by Dr. Alessandra Guarneri (René Rachou Institute, Fundação Oswaldo Cruz, Minas Gerais, Brazil), were maintained under the same conditions, with 20% FBS. Promastigotes of *Phytomonas serpens* 15 T strain were grown at 28 °C in GYPMI medium (glucose, yeast extract, peptone, and meat infusion) [45].

### 5.3. Extracellular Vesicles

Extracellular vesicles (EVs) were obtained from *T. cruzi* trypomastigotes (Vero cells supernatant), *T. cruzi* and *T. rangeli* epimastigotes, and *P. serpens* promastigotes. Parasites (at a concentration of 10^8^) were washed twice (1400× *g* for 10 min) and subjected to nutritional stress in 1 mL of RPMI without FBS for 2 h at 37 °C (for the trypomastigotes) or 28 °C (for the epimastigotes and promastigotes). After the incubation, the parasites were pelleted by centrifugation, and the supernatant was filtered (0.45 μm). The EVs were isolated from the supernatant by two rounds of ultracentrifugation at 100,000× *g* for 2 h at 4 °C. The EV pellets were resuspended in the residual phosphate-buffered saline (PBS; Lonza) from the centrifugation. The PBS from the nutritional stress supernatant without parasites was termed the ultracentrifugation supernatant (sUC) and used as a negative control.

EVs from non-infected Vero cells were used as an unrelated EV control. Vero cell monolayers were subjected to the same nutritional stress and supernatant purification procedure as described above, following the recommendations of Théry et al. [46]. For ELISA tests, EVs from *T. cruzi* and *T. rangeli* epimastigotes and Vero cells were purified using the Total Exosome Isolation from Cell Culture Media reagent (Invitrogen, Waltham, MA, USA), according to the manufacturer’s instructions.

EV concentration was estimated using a Qubit 2.0 Fluorometer (Invitrogen). The size distribution of the EVs was measured using a NanoSight LM10 instrument (Malvern, Panalytical, Great Malvern, England), and data were analyzed using Nanoparticle Tracking Analysis (NTA) 3.1 software (Malvern, Panalytical, Great Malvern, England). Five 30-second videos from two independent samples were analyzed. EVs detected at wavelengths greater than 400 nm were excluded as they were considered aggregates.

### 5.4. Electron Microscopy

For scanning electron microscopy (SEM), 7 × 10^7^ parasites under nutritional stress were washed twice in sodium cacodylate buffer (0.1 M, pH 7.2), adhered to pretreated poly-L-lysine (Sigma-Aldrich, St. Louis, MO, USA) coverslips, and fixed for 1 h at room temperature in Karnovsky fixative solution. Post-fixation was performed for 1 h with 1% osmium tetroxide (Sigma-Aldrich, St. Louis, MO, USA). The coverslips were then washed, gradually dehydrated in ethanol, and dried using a critical point dryer (Leica EM CPD 300—Leica, Wetzlar, Germany). The samples were coated with 20 nm of gold (Leica EM ACE 200—Leica, Wetzlar, Germany) and observed under a JEOL JSM-6010 Plus LA scanning electron microscope operated at 20 kV.

For transmission electron microscopy (TEM), 10 μL of purified parasite EVs (corresponding to 2 × 10^9^ parasites or approximately 50 ng/μL of protein) were applied for 10 min at room temperature on copper grids previously coated with 0.5% Formvar film and treated to remove static with a ZeroStat 3 gun. The samples were fixed for 1 h in Karnovsky fixative solution, washed in sodium cacodylate buffer, and stained for 1 min with 5% uranyl acetate (Sigma-Aldrich, St. Louis, MO, USA) or 2% pyrophosphotungstic acid (PTA; Sigma-Aldrich, St. Louis, MO, USA) at room temperature. The samples were observed using a JEOL JSM-1400 Plus transmission electron microscope operated at 80 or 100 kV.

### 5.5. ELISA

Protein extracts from parasites and Vero cells were obtained by lysis in buffer (50 mM Tris-HCl pH 7.4, 150 mM NaCl, 0.3% sodium deoxycholate, 1% NP40, 1 mM EDTA and PMSF) for 1 h at 4 °C, followed by removal of debris. Nunc MaxiSorp plates (Thermo Scientific, Waltham, MA, USA) were coated with 100 or 500 ng of EVs from the *T. cruzi* or *T. rangeli* epimastigotes, Vero cells, or protein extracts from these parasites and cells in carbonate-bicarbonate buffer (0.05 M, pH 9.6) for 16 h at 4 °C in a humid atmosphere.

Plates were incubated with blocking buffer (5% milk in PBS containing 0.05% Tween-20) for 1 h, followed by incubation at 37 °C for 2 h with (1) serum from Swiss mice 28 days post-infection with *T. cruzi* or *T. rangeli* trypomastigotes (three mice per tested parasite; serum diluted 1:50 or 1:100 in blocking buffer) or (2) pooled serum from five chronic *T. cruzi*-infected patients having the cardiac form (diluted 1:500 in blocking buffer). Sera from uninfected mice and healthy human donors were used as negative controls. Mice sera were provided by Dr. Alessandra Guarneri (Instituto René Rachou, Fiocruz-MG, CEUA/FIOCRUZ ethics committee protocol LW-3/22), and the patient sera were provided by Dr. Iara José de Messias-Reason (Hospital das Clínicas, Universidade Federal do Paraná, CEP/HC-UFPR ethics committee protocol 1457.122/2007-06).

After each step, the plates were washed five times with PBS containing 0.05% Tween-20. The immune complexes were detected by adding a peroxidase-conjugated anti-total IgG (Invitrogen) antibody: 1:1000 dilution for mouse serum or 1:4000 dilution for human serum, incubated for 1 h in blocking buffer. The plates were washed again and incubated with TMB or OPD substrate solution for 10 min, followed by stopping the reaction with sulfuric acid. Absorbance was measured at 450 nm for TMB or 520 nm for OPD using a microplate reader (Synergy H1 Hybrid, BioTek, Winooski, VT, USA). The obtained values were corrected by subtracting the blank value (reaction without antigen) for analysis.

### 5.6. Mass Spectrometry-Based Proteomic Analysis (LC-MS/MS)

Proteomic analysis of the EVs from the *T. rangeli* epimastigotes, *P. serpens* promastigotes, and *T. cruzi* trypomastigotes was performed according to the method described by Wowk et al. [47]. Three EV samples from each organism (5 µg of protein per sample) were denatured for 5 min at 95 °C in sample buffer (40 mM Tris-HCl, pH 6.8, 1% SDS, 2.5% β-mercaptoethanol, 6% glycerol, and 0.005% bromophenol blue). Protein extracts were loaded onto a 13% SDS-PAGE gel, which was stained with Coomassie blue. The lanes were excised and cut into 1 mm × 1 mm pieces, which were washed in 50% ethanol and 50 mM ammonium bicarbonate (pH 8.0), followed by dehydration in 100% ethanol for 10 min at 25 °C. The gel pieces were then dried for 7 min in a low-pressure centrifuge and rehydrated with 10 mM DTT and 50 mM ammonium bicarbonate (ABC) for 1 h at 56 °C. After this, they were incubated with 55 mM iodoacetamide and 50 mM ABC for 45 min at 25 °C, protected from light. The gel pieces were then washed and dehydrated twice with 50 mM ABC for 20 min, followed by dehydration in ethanol for 10 min, repeating the process. Next, the gel pieces were rehydrated in 12.5 ng/µL trypsin (Promega, Madison, WI, USA) and 50 mM ABC, and protein digestion was carried out at 37 °C for 16 h.

For peptide extraction, the supernatants were collected into new tubes. The gel was then incubated twice with extraction solution (3% TFA, 30% acetonitrile), followed by two additional incubations with 100% acetonitrile (ACN), each lasting 10 min at 25 °C. The supernatants from these incubations were pooled with the digestion supernatant. The final supernatant containing the digested peptides was completely dried using a low-pressure centrifuge (with vacuum).

Before submitting the samples for LC-MS/MS analysis, the peptides were purified using a C18 StageTip to desalinate the samples, and they were resuspended in 0.1% formic acid and 5% DMSO for injection into the mass spectrometer. Following these treatments, the samples were analyzed using the Thermo Scientific Easy-nLC 1000 ultra-performance liquid chromatography system coupled with the LTQ-Orbitrap XL ETD mass spectrometer at the Mass Spectrometry Facility RPT02H, Instituto Carlos Chagas/Fiocruz-PR. The peptide mixtures were loaded in triplicate onto an analytical column (15 cm long, 75 µm internal diameter, with 3 µm C18 particles) and eluted with a flow rate of 250 nL/min in a linear gradient from 5% to 40% acetonitrile, 0.1% formic acid, and 5% DMSO over 120 min. The mass spectrometer was set to acquire MS1 spectra in the m/z range of 300–2000 with a resolution of R = 60,000. For MS2, the 10 most intense peaks were selected for fragmentation, with dynamic exclusion set to 90 s.

All the mass spectra were analyzed using MaxQuant (version 2.2.0.0) with the NCBI database for *Phytomonas* (downloaded on 23 December 2022) and the TriTrypDB database (version 61) for *T. cruzi* Dm28c 2018 and *T. rangeli* SC58, applying a 1% FDR threshold for both spectrum and protein identification. Potential contaminants, reverse identifications, and peptides identified only by site were excluded. Downstream analysis included only the proteins that had at least one unique peptide identified in at least two biological replicates per microorganism. The quantification options LFQ and iBAQ were enabled. The mass spectrometry proteomics data have been deposited in the ProteomeXchange Consortium via the PRIDE partner repository [48] with the dataset identifier PXD040019 (https://dx.doi.org/10.6019/PXD040019: accessed on 21 January 2025).

### 5.7. Assignment of Proteins Domains and Orthologs

Proteins identified with at least two peptides in two biological replicates were considered for this analysis. The prediction of protein epitopes was based on their similarity to *T. cruzi* Dm28c 2018, using the Immune Epitope Database and Analysis Resource (IEDB). The epitope sequences provided by IEDB were mapped to the corresponding gene identifier in TriTrypDB using BLAST (similarity ≥ 97%). The confidence levels for each epitope set were classified as high or medium, based on the quality of the mapping.

Transmembrane domains were predicted using TMHMM2. The *Phytomonas* database from NCBI was annotated using the Blast2GO basic platform (version 6.0) [49]. Orthologs between the three species were identified using blastP (NCBI), with *T. cruzi*-identified proteins as the query and *T. rangeli*- or *P. serpens*-identified proteins as the subject. Hits that met the criteria of ≥70% identity and ≥70% match length (for both query and subject) were considered orthologs. All positive hits and proteins without hits were manually verified using protein descriptions from the respective protein databases (*T. cruzi* and *T. rangeli*) or annotations (*P. serpens*). For proteins with multiple hits, the one with the highest identity percentage was retained, while the others were discarded.

### 5.8. Extracellular Vesicles In Vitro Stimulation

PBMCs (2 × 10^5^ cells) or mdDCs (1 × 10^5^ cells/200 µL of media) were stimulated at 37 °C for 24 or 48 h with 1000 ng of EVs from the *T. cruzi* trypomastigotes, *T. rangeli* epimastigotes, or *P. serpens* promastigotes. As controls, cells were either left unstimulated (medium) or were stimulated with an equivalent volume of sUC, 100 ng/mL of *Escherichia coli* lipopolysaccharide (LPS-EK, InvivoGen, San Diego, CA, USA), or 1000 ng of Vero cell-derived EVs. After the stimulation period, cells were harvested and analyzed by flow cytometry.

In a separate experiment, after 24 h of stimulation with EVs, mdDCs were infected with *T. cruzi* trypomastigotes labeled with 0.1 mM CFSE (Sigma-Aldrich, St. Louis, MO, USA) at a multiplicity of infection (MOI) of 10. After 1 h of infection, non-internalized parasites were washed off, and the infection was assessed 16 h post-infection by flow cytometry.

### 5.9. Flow Cytometry

Cytokines in PBMC culture supernatants were quantified using the Human Inflammatory Cytokines Kit (BD Biosciences, Franklin Lakes, NJ, USA) according to the manufacturer’s instructions. For surface marker staining, PBMCs and mdDCs were detached using a cell scraper, washed with PBS, and blocked with 5% FBS and 1% human AB serum in PBS for 20 min at room temperature. The cells were then incubated for 20 min at room temperature with fluorochrome-conjugated mouse anti-human monoclonal antibodies specific for CD11b-APC-Cy7 (clone ICRF44; BD Biosciences), CD11c-PE-Cy5 (clone 3.9; BioLegend, San Diego, CA, USA), CD14-V450 (clone MΦP9; BD Biosciences), CD80/B7-1-PE-Cy7 (clone 2D10.4; BioLegend), and HLA-DR-APC-H7 (clone L243; BD Biosciences). After incubation, the cells were washed twice, and flow cytometry was performed on a FACS Canto II with BD FACSDiva software version 9.0 (BD Biosciences). The acquired data, comprising 50,000 events from each culture well, were analyzed using FlowJo v10 (BD Biosciences). Single-color-stained samples were used for instrument compensation, and control isotypes were also tested. Selective analysis of monocytes or mdDCs was performed by establishing single-cell gating, along with specific forward- versus side-scatter gating, and positive staining for the CD14^+^/CD11b^+^ and CD11c^+^/HLA-DR^+^, respectively. CD80 and CFSE levels were evaluated from the double-positive cell gate.

### 5.10. Mice Challenge

BALB/c mice were obtained from the Instituto Carlos Chagas/Fiocruz-PR animal facility and were maintained and handled according to the guidelines outlined in the Guide for the Care and Use of Laboratory Animals by the Brazilian National Council for Animal Experimentation. The Ethics Committee on the Use of Animals at Fundação Oswaldo Cruz approved the protocols (CEUA/FIOCRUZ, LW-61/12). Male mice, aged 8–12 weeks, 3 to 5 animals per group, were used in two independent experiments. The mice were inoculated intraperitoneally with EVs derived from 10^6^ *T. cruzi* cell-culture-derived trypomastigotes, *T. rangeli* epimastigotes, or *P. serpens* promastigotes, with four immunizations administered every 7 days. The control animals received only PBS. Seven days after the final immunization, the mice were challenged intraperitoneally with 10^5^ blood trypomastigotes of *T. cruzi*. Parasitemia was measured on day 3 post-infection (dpi) by collecting 5 µL of fresh blood from the tail vein [50]. Blood samples were collected every other day for 17 days, and survival was monitored for up to 30 days.

### 5.11. Statistical and Data Analysis

Analyses were conducted using GraphPad Prism 8 (GraphPad Prism 8 Software, Inc., Boston, MA, USA). Multiple *t*-tests were applied to compare the ELISA results, using the Benjamini–Hochberg False Discovery Rate (FDR) method with Q-1%. The Wilcoxon matched-pairs signed-rank test (nonparametric paired t-test) was used for analyzing the in vitro experiments with primary human cells to assess individual patterns. One-way ANOVA with Tukey’s multiple comparison test was employed for the animal experiments to compare group means, after passing the Shapiro–Wilk normality test and Bartlett’s test for homogeneity of variances. A *p*-value of <0.05 was considered statistically significant. Gene Ontology (GO) term enrichment was performed using the TritrypDB or Blast2GO platforms, with the respective species-specific database as the background. For the enrichment analysis of shared proteins, the *T. cruzi* database was used. GO terms with a Fisher’s Exact test *p*-value < 0.05 were considered significantly enriched. Venn diagrams were generated using the Venny 2.1 online tool [51].

## Figures and Tables

**Table 1:** List of EV proteins shared between *T. cruzi* trypomastigotes, *T. rangeli* epimastigotes, and *P. serpens* promastigotes.

*T. cruzi* ID	*T. rangeli* ID	*P. serpens* ID	Ortholog Group	Protein Description
Proteins common to *T. cruzi* and *T. rangeli* EVs				
C4B63_231g29	TRSC58_06706	-	-	flagellar calcium-binding protein
C4B63_50g201	TRSC58_04178	-	-	surface protease GP63 *
C4B63_18g171	TRSC58_01540	-	-	ATP synthase, epsilon chain
C4B63_44g221	TRSC58_06262	-	-	cytoskeleton-associated protein CAP5.5
C4B63_55g287c	TRSC58_06911	-	-	cytochrome c
C4B63_43g126	TRSC58_05688	-	-	ATP synthase F1 subunit gamma protein
C4B63_52g93	TRSC58_07087	-	-	trans-sialidase *^#^
C4B63_26g286	TRSC58_00214	-	-	arginine kinase
Proteins common to *T. cruzi*, *T. rangeli*, and *P. serpens* EVs				
C4B63_13g157	TRSC58_06854	CCW59913.1	OG6_100212	elongation factor 2
C4B63_351g33c	TRSC58_03454	CCW64249.1	OG6_148248	calpain-like cysteine peptidase
C4B63_295g22	TRSC58_02563	CCW65611.1	OG6_100111	tryparedoxin peroxidase
C4B63_47g72	TRSC58_07150	CCW64268.1	OG6_100260	enolase
C4B63_51g126	TRSC58_03588	CCW60849.1	OG6_100108	alpha tubulin
C4B63_97g34	TRSC58_04120	CCW64916.1	OG6_100641	P-type H+-ATPase *
Proteins common to *T. cruzi* and *P. serpens*				
C4B63_4g422	-	CCW60828.1	-	C-terminal motor kinesin
C4B63_19g183	-	CCW63407.1	-	cyclophilin a
C4B63_292g78c	-	CCW64575.1	-	calmodulin
Proteins common to *T. rangeli* and *P. serpens* EVs				
-	TRSC58_02653	CCW64813.1	OG6_100712	adenylate kinase
-	TRSC58_01995	CCW63656.1	OG6_111169	trypanothione synthetase
-	TRSC58_03782	CCW67782.1	OG6_100076	ABC transporter
-	TRSC58_04008	CCW64170.1	OG6_101294	eukaryotic initiation factor 4a
-	TRSC58_03503	CCW64848.1	OG6_100294	threonyl-tRNA synthetase
-	TRSC58_03680	CCW62159.1	OG6_100353	cystathione gamma lyase
-	TRSC58_04758	CCW61177.1	OG6_100715	S-adenosylhomocysteine hydrolase
	TRSC58_06901	CCW63810.1	OG6_100418	hexokinase
-	TRSC58_01051	CCW63217.1	OG6_103540	2,3-bisphosphoglycerate-independent phosphoglycerate mutase

* Protein with transmembrane domain. ^#^ Protein with epitope sequence described.

**Table 2:** Proteins with predicted transmembrane domains (TMD) and/or epitope sequences in trypanosomatid EVs.

spp.	ID	Protein Description	TMD	Epitopes
*T. rangeli*	TRSC58_02343	hypothetical protein	14	na
	TRSC58_06436	hypothetical protein	9	na
	TRSC58_04120	P-type H+-ATPase *	7	na
	TRSC58_00699	hypothetical protein	6	na
	TRSC58_07060	40S ribosomal protein S3a	2	na
	TRSC58_00147	hypothetical protein	1	na
	TRSC58_04043	aspartate aminotransferase, mitochondrial	1	na
	TRSC58_04178	surface protease GP63 *	1	na
	TRSC58_04835	chaperonin GroEL	1	na
	TRSC58_05116	calreticulin	1	na
	TRSC58_07191	hypothetical protein	1	na
*T. cruzi*	C4B63_44g146	vacuolar proton pyrophosphatase 1	16	5
	C4B63_49g193	trans-sialidase, Group II	3	3
	C4B63_26g29	trans-sialidase, Group I	1	2
	C4B63_52g93	trans-sialidase *	1	1
	C4B63_43g9	serine carboxypeptidase (CBP1)	1	na

* Protein shared in EVs of at least 2 species.

## Data Availability

The mass spectrometry proteomics data have been deposited in the ProteomeXchange Consortium via the PRIDE partner repository with the dataset identifier PXD040019 (https://dx.doi.org/10.6019/PXD040019: 21 January 2025).

## Supplementary Materials

The following supporting information can be downloaded at https://www.mdpi.com/article/10.3390/ijms26041544/s1.
