# Supplementary material for: Trypanosomatid Extracellular Vesicles as Potential Immunogens for Chagas Disease
Source: Int J Mol Sci. 2025 Feb 12;26(4):1544. doi: 10.3390/ijms26041544 (PMC11855489; doi:10.3390/ijms26041544)
Supplement: Supplementary file 1 [file ijms-26-01544-s001.zip › ijms-3428940-supplementary.pdf]

International Journal of
Molecular Sciences

MDPI

Supplementary material

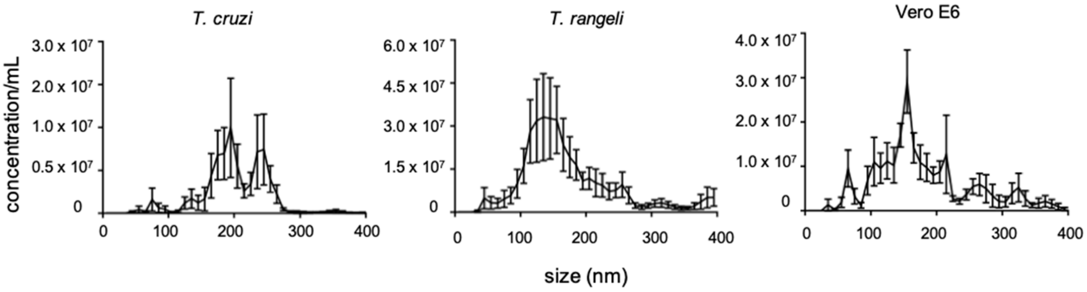

Figure S1. Extracellular vesicles (EVs) of *T. cruzi, T. rangeli* and Vero E6 cells. EVs were extracted by commercial kit (Invitrogen Total Exosome Isolation from Cell Culture Media reagent). Concentration and size distribution ± standard error of the mean (SEM) are shown of EVs from *T. cruzi* and *T. rangeli* epimastigote forms and Vero E6 cells. Data obtained from Nanoparticle Tracking Analysis (NTA) of five 30-second videos from two independent samples.

Table S1. List of all identified proteins in extracellular vesicles (EVs) from *P. serpens* promastigotes and *T. rangeli* epimastigotes. Data are derived from LC-MS/MS analysis of three independent EV samples from each trypanosomatid (5 μg of protein per sample). The downstream analysis included only proteins with at least one unique peptide identified in at least two biological replicates per microorganism.

***P. serpens* EV**	***T. rangeli* EV**
elongation factor 2	elongation factor 2
calpain-like cysteine peptidase	calpain-like cysteine peptidase
tryparedoxin peroxidase	tryparedoxin peroxidase
enolase	enolase
alpha tubulin	alpha tubulin
P-type H+-ATPase	P-type H+-ATPase
trypanothione synthetase	trypanothione synthetase
ABC transporter	ABC transporter
eukaryotic initiation factor 4a	eukaryotic initiation factor 4a
threonyl-tRNA synthetase	threonyl-tRNA synthetase
S-adenosylhomocysteine hydrolase	S-adenosylhomocysteine hydrolase
cystathione gamma lyase	cystathione gamma lyase
hexokinase	hexokinase
adenylate kinase	adenylate kinase
2,3-bisphosphoglycerate-independent phosphoglycerate mutase	2,3-bisphosphoglycerate-independent phosphoglycerate mutase
C-terminal motor kinesin	flagellar calcium-binding protein
cyclophilin a	surface protease GP63
calmodulin	ATP synthase, epsilon chain
ubiquitin-conjugating enzyme	cytoskeleton-associated protein CAP5.5
Rab other	cytochrome c
proteasome alpha 7 subunit	ATP synthase F1 subunit gamma protein
glucose-regulated 78	trans-sialidase
small GTP-binding	arginine kinase
Proteasome regulatory non-ATPase subunit	hypothetical protein
arginyl-tRNA synthetase	60S ribosomal protein L7
alpha-soluble NSF attachment	succinate dehydrogenase flavoprotein
Clathrin heavy chain	40S ribosomal protein S16
pyruvate kinase	glucokinase 1
paraflagellar rod 1D	14-3-3 protein
UDP-sugar pyrophosphorylase	peptidase M20/M25/M40
tyrosine phosphatase	pyridoxal kinase
nitrilase	cytosol alanyl aminopeptidase
paraflagellar rod 2C	lipophosphoglycan biosynthetic protein
proteasome alpha 3 subunit	alcohol dehydrogenase
chaperonin mitochondrial precursor	40S ribosomal protein S4
hypersensitive-induced response 1-like	succinyl-coA:3-ketoacid-coenzyme A transferase, mitochondrial precursor

fatty acyl synthetase 2	fatty acyl CoA synthetase
glycosomal membrane	L-threonine 3-dehydrogenase
rab-GDP dissociation inhibitor	solute carrier family 25 (mitochondrial adenine nucleotide translocator)
proteasome regulatory non-ATP-ase subunit	aminopeptidase
GDP-mannose pyrophosphorylase	glyceraldehyde 3-phosphate dehydrogenase, cytosolic
NA	phosphoglycerate kinase
unnamed protein product	ATP-dependent Clp protease subunit, heat shock protein 78
Proteasome beta 6 subunit	mitochondrial tryparedoxin peroxidase
proteasome regulatory ATPase subunit 5	branched-chain amino acid aminotransferase
metallo- Clan MA(E) Family M32	cytosolic malate dehydrogenase
translation elongation factor 1-beta	nucleoside diphosphate kinase
heat shock partial	glycosomal malate dehydrogenase
heat shock 83-1	phosphoenolpyruvate carboxykinase (ATP)
alanyl-tRNA synthetase	glyceraldehyde 3-phosphate dehydrogenase
phosphomannomutase	protein disulfide isomerase
26S proteasome regulatory subunit	tyrosine aminotransferase
aspartyl-tRNA synthetase	heat shock 70kDa protein 1/8
vesicle-associated membrane	fatty acyl CoA synthetase 2
vesicle-fusing ATPase	quinone oxidoreductase
chaperonin alpha subunit	chaperonin GroEL
proteasome alpha 5 subunit	tryparedoxin
proteasome regulatory ATPase subunit 2	malate dehydrogenase
amino acid transporter	carboxypeptidase Taq
stress-induced sti1	aspartate aminotransferase
phosphatidic acid phosphatase	fructose-bisphosphate aldolase, glycosomal
Thiol-dependent reductase 1	molecular chaperone HtpG
prostaglandin f synthase	eukaryotic initiation factor 5a
small GTP-binding Rab7	10 kDa heat shock protein
conserved SNF-7	calreticulin
mitochondrial small myristoylated - (SMP-1)	triosephosphate isomerase
asparagine synthetase A	D-isomer specific 2-hydroxyacid dehydrogenase-protein
udp-glc 4 -epimerase	glucose-regulated protein 78
UDP-glucose 6-dehydrogenase 1-like	elongation factor 1-alpha (EF-1-alpha)
proteasome beta 5 subunit	2-amino-3-ketobutyrate coenzyme A ligase
14-3-3.	ATP synthase alpha chain, mitochondrial precursor
kinetoplastid membrane KMP-11	ATPase beta subunit
chaperone DNAj	pyruvate phosphate dikinase
Proteasome beta-1 subunit	tubulin beta
intraflagellar transport	nucleoside transporter
rab1 small GTP-binding	sterol 24-c-methyltransferase
vacuolar sorting-associated 4	kinesin-like protein

sodium stibogluconate resistance	succinyl-CoA ligase [GDP-forming] beta-chain
ADP-ribosylation factor-like small GTPase	thiol-dependent reductase 1
cystathionine beta-synthase	citrate synthase
malic enzyme	seryl-tRNA synthetase
Proliferative cell nuclear antigen (PCNA)	prostaglandin F2alpha synthase
glutathione peroxidase	glutaredoxin
flagellar associated	40S ribosomal protein S3a
asparaginyl-tRNA synthetase	glycerol dehydrogenase
mitochondrial long-chain-fatty-acid- ligase	heat shock 70kDa protein 4
trypanothione reductase	ADP-ribosylation factor 3
elongation factor 1- partial	actin
ADP ribosylation factor 3	heat shock 70 kDa protein, mitochondrial precursor
Transitional endoplasmic reticulum ATPase	aspartate aminotransferase, mitochondrial
stress-inducible STI1	NADH-dependent fumarate reductase
ubiquitin-fusion	
ADP-ribosylation factor	
small glutamine-rich tetratricopeptide repeat	
regulator of microtubule dynamics 1	
Proteasome alpha 2 subunit	
cytosolic NADP-malic partial	
fatty acyl syntetase 1	
antigenic WD	
nucleoside diphosphate kinase B	
dimethylargininase	
mitochondrial heat-shock hsp70	
membrane associated	
Heat shock	
indolepyruvate decarboxylase	

Table S2. Description of proteins with predicted epitope sequences identified in trypanosomatid extracellular vesicles. The epitope prediction was based on sequence similarity to *T. cruzi* Dm28c 2018, utilizing the Immune Epitope Database and Analysis Resource (IEDB). The epitope sequences provided by IEDB were mapped to the corresponding gene identifier in TriTrypDB using BLAST, with a similarity threshold of ≥ 97%).

***T. cruzi* ID**	**Protein description**	**Epitope sequence**	**Location**	**Confidence**
C4B63_133g58	heat shock protein 70 (hsp70)	155917 TIAGMEVLRI	164-173	medium
C4B63_133g58	heat shock protein 70 (hsp70)	156212 YVAFTDTERL	41-50	medium
C4B63_133g58	heat shock protein 70 (hsp70)	155548 QVAMNPTNTV	58-67	medium
C4B63_24g328	trans-sialidase, Group II	68051 VDQNFTLV	552-559	medium
C4B63_24g328	trans-sialidase, Group II	37352 LLGMWGIAAI	735-744	medium
C4B63_24g328	trans-sialidase, Group II	98126 ITATIEGRK	377-385	medium
C4B63_26g29	trans-sialidase, Group I	174282 STPVDSSAHGTPSTP	678-692	medium
C4B63_26g29	trans-sialidase, Group I	3304 ANHAFTLV	510-517	medium
C4B63_49g193	trans-sialidase, Group II	18144 FVDYNFSLV	613-621	medium
C4B63_49g193	trans-sialidase, Group II	37342 LLGLWGFAAL	773-782	medium
C4B63_49g193	trans-sialidase, Group II	68101 VDYNFSLV	614-621	medium
C4B63_52g93	trans-sialidase	3335 ANKRFTLV	552-559	medium
C4B63_62g93	unspecified product	18144 FVDYNFSLV	550-558	medium
C4B63_62g93	unspecified product	37342 LLGLWGFAAL	717-726	medium
C4B63_62g93	unspecified product	68101 VDYNFSLV	551-558	medium
C4B63_84g89	heat shock protein 85	8902 DKIRYQSL	42-49	medium
